# Multimorbidity of hidradenitis suppurativa: a cross-sectional population-based study of its associated comorbidities

**DOI:** 10.3389/fmed.2025.1618975

**Published:** 2025-09-05

**Authors:** Manuel Almenara-Blasco, Tamara Gracia-Cazaña, Beatriz Poblador-Plou, Clara Laguna-Berna, Alba Navarro-Bielsa, Aida Moreno-Juste, Antonio Gimeno-Miguel, Yolanda Gilaberte

**Affiliations:** ^1^Servicio de Dermatología, Hospital Universitario Miguel Servet, Zaragoza, Spain; ^2^EpiChron Group for Research in Chronic Diseases, Aragón Health Research Institute, Zaragoza, Spain; ^3^Research Network on Chronicity, Primary Care and Health Promotion (RICAPPS), Barcelona, Spain

**Keywords:** hidradenitis suppurativa, comorbidities, Epidemiology, population-based study, multimorbidity

## Abstract

**Background:**

Hidradenitis suppurativa (HS) is a chronic, inflammatory skin disorder associated with significant physical and psychological burden. While individual comorbidities have been described in prior studies, a comprehensive analysis of all chronic conditions associated with HS in a large population-based cohort is lacking.

**Objectives:**

To determine the prevalence of hidradenitis suppurativa in a large-scale, population-based study, to describe its comorbidities, and to analyze which diseases are associated with the presence of hidradenitis.

**Methods:**

Retrospective cross-sectional observational study based on the clinical information contained in the electronic health records from the individuals of the EpiChron Cohort (Aragón, Spain) with a diagnosis of hidradenitis suppurativa (1,003 individuals from all ages and sexes) in 2019. We analyzed the prevalence of chronic comorbidities and used logistic regression models adjusted for age, sex and comorbidities to calculate the likelihood of occurrence of each of the comorbidities with a prevalence ≥1% based on the presence of hidradenitis. We used a cut-off point for statistical significance of *p*-value < 0.05.

**Results:**

The prevalence of hidradenitis suppurativa was 0.10%, and it was more prevalent in women (0.12% vs. 0.08%). The most frequent chronic comorbidities were disorders of lipid metabolism (27.8%), hypertension (19.7%), and anxiety disorders (18.7%). The conditions most associated with hidradenitis were (odds ratio; 95% confidence interval) skin and subcutaneous tissue infections (3.32; 2.56–4.30), diseases of white blood cells (2.11; 1.44–3.07), and schizophrenia and other psychotic disorders (2.17; 1.16–4.06), among others.

**Conclusion:**

HS is associated with a high burden of multimorbidity, encompassing metabolic, neuropsychiatric, musculoskeletal, and infectious conditions. These findings underscore the need for integrated and multidisciplinary management strategies. Further longitudinal research is warranted to explore causal relationships and improve clinical outcomes.

## 1 Introduction

Hidradenitis suppurativa (HS) is a chronic, inflammatory skin disease that primarily affects intertriginous areas such as the axillae, groin, and anogenital region, where apocrine glands are concentrated (1, 2). It is characterized by recurrent, painful nodules, abscesses, sinus tracts, and scarring. Diagnosis is typically clinical, often utilizing the Hurley staging system to assess severity. Onset usually occurs in early adulthood, and HS can persist for decades, significantly impacting quality of life (1, 2). Global prevalence estimates vary widely (0.00033–4.1%) due to differences in diagnostic criteria, populations studied, and methodologies. Highest rates of HS are reported in Europe (1.4%), followed by North America (0.67%) and Asia (0.42%). HS is more common in women, with reported female-to-male ratios ranging from 1.5:1 to 4:1 (3–5).

Beyond its dermatologic manifestations, HS is increasingly recognized as a systemic condition associated with a wide range of comorbidities that affect multiple organ systems (2, 6, 7). Research has linked HS to various other illnesses, and demonstrated a strong association with components of metabolic syndrome, including obesity, dyslipidemia, hypertension, and type 2 diabetes mellitus (6–9). These conditions are significantly more prevalent in HS patients than in the general population: some studies have reported metabolic syndrome in up to 65% of individuals with HS, versus an estimated 10–25% in the general population (6, 7, 10). Obesity in particular is strongly linked to HS, potentially contributing to its pathogenesis through mechanical stress in skin folds and chronic systemic inflammation. Lipid abnormalities and insulin resistance may also exacerbate disease severity and progression (8, 10).

HS is associated with increased cardiovascular risk (11, 12). Elevated rates of hypertension, cerebrovascular disease, and peripheral vascular disorders have been observed in HS patients, potentially contributing to higher cardiovascular mortality (11, 12). Some studies estimate a 1.7-fold increased risk of cardiovascular-related death in individuals with HS (11, 12). While the independent contribution of HS to cardiovascular risk remains unclear, the frequent co-occurrence of metabolic comorbidities underscores the need for early screening and intervention (10, 13–15).

Gastrointestinal (GI) comorbidities, particularly inflammatory bowel diseases (IBD) such as Crohn’s disease and ulcerative colitis, also more prevalent among patients with HS (16). The mechanisms linking HS and IBD remain poorly understood, but may involve shared genetic susceptibility, dysregulated immune responses, and overlapping environmental risk factors (16). Although reported prevalences vary widely, epidemiological studies reveal a several-fold increased risk of IBD in HS patients, with some case–control studies estimating a 10-fold higher risk of Crohn’s disease (2, 6, 16).

Musculoskeletal comorbidities, especially spondyloarthritis (including ankylosing spondylitis and psoriatic arthritis), are also associated with HS (17). These inflammatory joint disorders are often underdiagnosed but can significantly impair function and quality of life. Prevalence estimates of spondyloarthritis in HS patients range from 1.5 to 40% (17), but rarely exceed 2% in the general population (18). Shared inflammatory pathways, including elevated levels of TNF-*α* and IL-17, may explain this overlap (17, 18).

Neuropsychiatric comorbidities are common in HS and substantially contribute to disease burden (19). The chronic pain, visible lesions, malodor, and stigma associated with HS likely contribute to the psychological distress experienced by patients. Recent studies have highlighted a possible association between HS and more severe psychiatric conditions such as schizophrenia and other psychotic disorders, potentially pointing to shared inflammatory mechanisms, including systemic cytokine dysregulation (9, 20–22). Prevalence rates of depression (15–65%) and anxiety (13–36%) in HS patients are also significantly higher than those reported in the general population (20–22).

Infectious diseases constitute another important comorbidity category in HA patients, who are at increased risk of secondary skin and soft tissue infections due to impaired barrier function, recurrent abscess formation, and colonization by pathogenic bacteria (1, 2, 9). The increased prevalence of mycoses and chronic skin infections in HS patients can potentially complicate disease management and therapeutic choices (2, 23).

A better understanding of the comorbid profile of HS is essential to improve care. While previous studies have explored individual comorbidities, few have comprehensively evaluated the full spectrum of chronic conditions in a large, unselected population. In this study, we sought to (i) estimate the prevalence of HS in a large population-based cohort in Aragón, Spain; (ii) describe the sociodemographic and clinical characteristics of affected individuals; and (iii) identify chronic comorbidities that are significantly associated with HS.

## 2 Methods

### 2.1 Study design, setting and participants

This retrospective cross-sectional study was conducted using the EpiChron Cohort in Aragón, Spain. This database integrates sociodemographic and clinical information from all users of the Aragonese public health system (population 1.3 million; approximately 98% public health system utilization) with at least one chronic condition recorded between 2010 and 2019. The study population included 1,098,383 individuals with chronic conditions, of whom 1,003 had a diagnosis of HS in 2019. HS diagnosis was determined by the presence of an active clinical episode with ICD-9-CM code 705.83 in their electronic health records (EHRs). Individuals without this code served as comparators. Individuals not registered in the health system in 2018, precluding retrospective comorbidity assessment, were excluded. The study was approved by the Clinical Research Ethics Committee of Aragón (CEICA; PI23/607). Due to the epidemiological nature and use of anonymized data, informed consent was waived.

### 2.2 Study variables and data sources

For each participant, we analyzed sex, age, country of birth, area of residence (rural vs. urban), area-level deprivation index (categorized into quartiles, Q1–Q4, from least to most deprived) (24), smoking status, and all chronic conditions documented in their EHRs. Diagnoses were initially coded using ICPC-1 and mapped to ICD-9-CM codes. Each ICD-9-CM code was then categorized into 226 mutually exclusive clinical categories using the Clinical Classifications Software (CCS) (25). Of these, 153 were classified as chronic using the Chronic Condition Indicator (CCI) open source tool (26) which defines chronic conditions as those lasting ≥ 12 months and meeting at least one of the following criteria: (a) requiring continuous care with high recurrence risk and/or implications for patient management; or (b) causing limitations in self-care, independent living, or social interaction. Demographic variables were retrieved from the user database with mandatory completion, ensuring complete data for all participants. The absence of a registered diagnostic code was considered the absence of the disease, not missing data.

### 2.3 Statistical analysis

We calculated the prevalence of HS and described its distribution across sociodemographic variables. Logistic regression models were used to estimate crude and age- and sex-adjusted odds ratios (ORs) for HS based on these variables. To identify comorbidities associated with HS, we used logistic regression models to calculate the likelihood of each comorbidity (dependent variable) based on the presence of HS (independent variable). Two models were generated for each comorbidity: one unadjusted (crude OR) and one adjusted for age, sex, and all other comorbidities. Smoking status and obesity were not included in the adjusted models due to underreporting in the database. Statistical significance was set at *p* < 0.05. Analyses were performed using RStudio.

## 3 Results

### 3.1 Prevalence and socio-demographic features of hidradenitis suppurativa

A total of 1,003 patients had a diagnosis of HS during the study period (mean age 40.54 years, SD 16.47; 63.41% women), resulting in an overall prevalence of 0.10% (Table 1). This prevalence was significantly higher in women than men (0.12% vs. 0.08%; OR 1.52, 95% CI 1.34–1.73; Table 1). Compared to Spanish individuals, the prevalence of HS was higher among Sub-Saharan Africans (aOR 2.53; 95% CI 1.68–3.80), Latin Americans (aOR 1.59; 95% CI 1.27–2.00), and North Africans (aOR 2.00; 95% CI 1.43–2.79). Asian, North American, and Eastern European individuals had similar prevalence to Spanish individuals (Table 2). HS was comparably prevalent in rural and urban areas (0.10% vs. 0.10%; aOR 0.99, 95% CI 0.87–1.12), and more prevalent in the most deprived areas (Q4) (0.12%; aOR 1.43, 95% CI 1.21–1.69). No significant differences in prevalence were observed between Q1, Q2, and Q3 (Table 2).

**Table 1:** Prevalence of hidradenitis suppurativa in the EpiChron Cohort (Aragon, Spain) in 2019 based on sociodemographic variables.

Hidradenitis prevalence	Men (*n* = 509,506)	Women (*n* = 588,877)	Total (*n* = 1,098,383)
	*n* (%)	*n* (%)	*n* (%)
Sex	367 (0.08)	636 (0.12)	1,003 (0.10)
Age (years)			
0–17	21 (0.03)	60 (0.10)	81 (0.06)
18–44	185 (0.14)	341 (0.22)	526 (0.18)
45–64	125 (0.08)	193 (0.12)	318 (0.10)
≥65	36 (0.03)	42 (0.03)	78 (0.03)
Nationality			
Spain	297 (0.07)	503 (0.11)	800 (0.09)
Eastern Europe	20 (0.11)	26 (0.11)	46 (0.11)
Asia	1 (0.04)	6 (0.22)	7 (0.13)
North Africa	12 (0.12)	24 (0.29)	36 (0.20)
Sub-Saharan Africa	9 (0.14)	15 (0.41)	24 (0.24)
Latin America	24 (0.14)	57 (0.18)	81 (0.17)
EU and North America	4 (0.09)	5 (0.11)	9 (0.10)
Area of residence			
Urban	211 (0.08)	402 (0.12)	613 (0.10)
Rural	156 (0.08)	234 (0.11)	390 (0.10)
Deprivation index^1^			
Q_1_	88 (0.07)	144 (0.10)	232 (0.09)
Q_2_	98 (0.08)	148 (0.11)	246 (0.10)
Q_3_	66 (0.07)	120 (0.11)	186 (0.09)
Q_4_	115 (0.09)	224 (0.15)	339 (0.12)

1 Deprivation index of the area of residence according to 26 socio-economic indicators and categorized from least (Q1) to most (Q4) deprived.

**Table 2:** Likelihood of presenting with hidradenitis suppurativa based on socio-demographic variables, calculated using logistic regression models.

Variable	Crude OR^1^	*p*-value	Adjusted OR^2^	*p*-value
Sex				
Men	ref.			
Women	1.52 (1.34–1.73)	0.000		
Age (years)				
0–17	0.35 (0.27–0.44)	0.000		
18–44	ref.			
45–64	0.54 (0.47–0.62)	0.000		
≥65	0.15 (0.12–0.19)	0.000		
Nationality				
Spain	ref.		ref.	
Sub-Saharan Africa	2.65 (1.77–3.98)	0.000	2.53 (1.68–3.80)	0.000
Asia	1.43 (0.68–3.02)	0.342	1.25 (0.59–2.62)	0.564
Eastern Europe	1.23 (0.91–1.66)	0.170	1.09 (0.81–1.47)	0.568
Latin America	1.84 (1.46–2.31)	0.000	1.59 (1.27–2.00)	0.000
North Africa	2.17 (1.55–3.03)	0.000	2.00 (1.43–2.79)	0.000
EU and North America	1.11 (0.57–2.14)	0.758	1.12 (0.58–2.16)	0.732
Area of residence				
Urban	ref.		ref.	
Rural	0.97 (0.86–1.10)	0.674	0.99 (0.87–1.12)	0.889
Deprivation index^3^				
Q_1_	ref.		ref.	
Q_2_	1.15 (0.96–1.37)	0.140	1.17 (0.97–1.40)	0.090
Q_3_	1.03 (0.85–1.25)	0.773	1.06 (0.88–1.29)	0.523
Q_4_	1.40 (1.18–1.65)	0.000	1.43 (1.21–1.69)	0.000

1 Odds ratio.

2 Adjusted odds ratios for sex and age.

3 Deprivation index of the area of residence according to 26 socio-economic indicators and categorized from least (Q1) to most (Q4) deprived.

### 3.2 Comorbidity of hidradenitis suppurativa

Among patients with HS, 70.99% exhibited multimorbidity, with a mean disease burden of 3.19 (SD 2.51) chronic conditions (Table 3). Approximately one-third of patients with HS had a documented history of smoking in their EHRs (Table 3).

**Table 3:** Socio-demographic and clinical characteristics of patients with hidradenitis suppurativa in the EpiChron Cohort in 2019.

Characteristics	Men	Women	Total
*N* (%)	367 (36.59)	636 (63.41)	1,003 (100)
Mean age, years (SD^1^)	42.15 (16.47)	39.61 (16.40)	40.54 (16.47)
Age group, years (*n*, %)			
0–17	21 (5.72)	60 (9.43)	81 (8.08)
18–44	185 (50.41)	341 (53.62)	526 (52.44)
45–64	125 (34.06)	193 (30.35)	318 (31.70)
≥65	36 (9.81)	42 (6.60)	78 (7.78)
Nationality (*n*, %)			
Spain	297 (80.93)	503 (79.09)	800 (79.76)
Eastern Europe	20 (5.45)	26 (4.09)	46 (4.59)
Asia	1 (0.27)	6 (0.94)	7 (0.70)
North Africa	12 (3.27)	24 (3.77)	36 (3.59)
Sub-Saharan Africa	9 (2.45)	15 (2.36)	24 (2.39)
Latin America	24 (6.54)	57 (8.96)	81 (8.08)
EU and North America	4 (1.09)	5 (0.79)	9 (0.90)
Area of residence^2^			
Urban (*n*, %)	211 (57.49)	402 (63.21)	613 (61.12)
Deprivation index^3^ (*n*, %)			
Q_1_	88 (23.98)	144 (22.64)	232 (23.13)
Q_2_	98 (26.70)	148 (23.27)	246 (24.53)
Q_3_	66 (17.98)	120 (18.87)	186 (18.54)
Q_4_	115 (31.34)	224 (35.22)	339 (33.80)
Smoking, yes^4^ (*n*, %)	128 (34.88)	184 (28.93)	312 (31.11)
Number of chronic diseases (mean, s.d.)	2.94 (2.44)	3.33 (2.54)	3.19 (2.51)
Multimorbidity, yes (*n*, %)	250 (68.12)	462 (72.64)	712 (70.99)

1 Standard deviation.

2 Urban versus rural.

3 Deprivation index of the area of residence according to 26 socio-economic indicators and categorized from least least (Q1) to most (Q4) deprived.

4 Retrieved from patient’s general descriptors between 2014 and 2019.

The most frequent chronic comorbidities in patients with HS were disorders of lipid metabolism (27.82%), hypertension (19.74%), and anxiety disorders (18.74%) (Table 4).

**Table 4:** Likelihood of comorbidities based on the presence or absence of hidradenitis suppurativa in the EpiChron Cohort.

Comorbidity	Prevalence	Crude OR	Adjusted OR^1^	*p*-value^2^
	*n* (%)	(95% CI)	(95% CI)	
Disorders of lipid metabolism	279 (27.82)	0.87 (0.76–1.00)	1.31 (1.08–1.59)	0.006
Hypertension	198 (19.74)	0.62 (0.53–0.72)	1.26 (1.05–1.52)	0.014
Anxiety disorders	188 (18.74)	1.37 (1.16–1.60)	1.19 (1.01–1.40)	0.036
Other nutritional; endocrine; and metabolic disorders	184 (18.34)	0.94 (0.80–1.10)	1.06 (0.86–1.31)	0.576
Spondylosis; intervertebral disc disorders; other back problems	183 (18.25)	1.29 (1.10–1.51)	1.34 (1.14–1.59)	<0.001
Headache; including migraine	165 (16.45)	1.79 (1.51–2.11)	1.36 (1.14–1.61)	<0.001
Menstrual disorders	163 (16.25)	2.19 (1.85–2.59)	1.35 (1.12–1.63)	0.001
Other non-traumatic joint disorders	151 (15.05)	1.45 (1.22–1.73)	1.31 (1.10–1.56)	0.003
Obesity	147 (14.66)	1.61 (1.35–1.92)	1.67 (1.39–2.01)	<0.001
Other upper respiratory disease	137 (13.66)	1.12 (0.93–1.34)	0.95 (0.79–1.13)	0.572
Thyroid disorders	133 (13.26)	1.17 (0.97–1.40)	1.19 (0.98–1.43)	0.076
Depression and mood disorders	128 (12.76)	1.01 (0.84–1.22)	1.07 (0.88–1.29)	0.517
Other ear and sense organ disorders	115 (11.47)	0.95 (0.78–1.16)	1.04 (0.85–1.26)	0.723
Blindness and vision defects	107 (10.67)	1.20 (0.98–1.46)	1.10 (0.90–1.34)	0.362
Allergic reactions	95 (9.47)	0.98 (0.79–1.21)	0.78 (0.63–0.96)	0.022
Other inflammatory condition of skin	93 (9.27)	1.78 (1.44–2.20)	1.66 (1.34–2.06)	<0.001
Inflammation; infection of eye (except that caused by tuberculosis or sexually transmitted disease)	78 (7.78)	1.57 (1.24–1.97)	1.41 (1.11–1.78)	0.004
Other connective tissue disease	76 (7.58)	1.30 (1.03–1.64)	1.13 (0.89–1.43)	0.323
Diabetes Mellitus	75 (7.48)	0.77 (0.61–0.98)	1.34 (1.04–1.74)	0.023
Asthma	74 (7.38)	1.03 (0.81–1.30)	0.90 (0.71–1.15)	0.405
Osteoarthritis	72 (7.18)	0.62 (0.49–0.79)	1.24 (0.95–1.61)	0.106
Residual codes; unclassified	72 (7.18)	1.25 (0.98–1.59)	1.40 (1.09–1.79)	0.008
Urinary tract infections	67 (6.68)	1.18 (0.92–1.52)	1.05 (0.82–1.36)	0.693
Skin and subcutaneous tissue infections	62 (6.18)	3.89 (3.00–5.03)	3.32 (2.56–4.30)	<0.001
Other lower respiratory disease	61 (6.08)	1.14 (0.88–1.48)	1.07 (0.82–1.39)	0.624
Other nervous system disorders	60 (5.98)	1.40 (1.08–1.82)	1.31 (1.01–1.72)	0.045
Neoplasms	59 (5.88)	0.99 (0.76–1.29)	1.24 (0.95–1.62)	0.113
Nutritional deficiencies	57 (5.68)	1.39 (1.06–1.81)	1.45 (1.10–1.90)	0.008
Other gastrointestinal disorders	54 (5.38)	1.61 (1.22–2.12)	1.48 (1.12–1.95)	0.006
Mycoses	53 (5.28)	2.22 (1.68–2.92)	1.67 (1.26–2.21)	<0.001
Genitourinary symptoms and ill-defined conditions	53 (5.28)	0.69 (0.52–0.90)	1.18 (0.88–1.58)	0.271
Menopausal disorders	53 (5.28)	1.37 (1.04–1.81)	1.37 (1.03–1.83)	0.033
Other eye disorders	49 (4.89)	1.27 (0.95–1.69)	1.25 (0.94–1.67)	0.127
Miscellaneous mental health disorders	47 (4.69)	1.57 (1.17–2.10)	1.17 (0.87–1.58)	0.294
Conditions associated with dizziness or vertigo	45 (4.49)	1.29 (0.96–1.74)	1.23 (0.91–1.66)	0.182
Nonmalignant breast conditions	42 (4.19)	1.97 (1.44–2.68)	1.43 (1.04–1.96)	0.027
Viral infection	40 (3.99)	1.45 (1.06–2.00)	1.24 (0.90–1.71)	0.185
Cardiac dysrhythmias	36 (3.59)	0.69 (0.49–0.96)	1.20 (0.84–1.70)	0.308
Other acquired deformities	33 (3.29)	1.34 (0.95–1.90)	1.11 (0.78–1.57)*	0.560

1 Odds ratios adjusted by sex, age and for the presence of the rest of comorbidities studied.

2 *p*-values <0.005 for the adjusted OR.

After adjusting for sex, age, and other comorbidities, the conditions most strongly associated with HS were skin and subcutaneous tissue infections (aOR 3.32; 95% CI 2.56–4.30), diseases of white blood cells (aOR 2.11; 95% CI 1.44–3.07), and schizophrenia and other psychotic disorders (aOR 2.17; 95% CI 1.16–4.06) (Table 4). Other significantly associated comorbidities included obesity, nutritional deficiencies, diabetes mellitus, disorders of lipid metabolism, hypertension, other gastrointestinal disorders, sexual disorders, anxiety disorders, spondylosis, and mycoses. Some comorbidities, including female infertility, asthma, coagulation and hemorrhagic disorders, and acute myocardial infarction, did not show a significant association with HS (See Table 4 for complete results). For osteoporosis and other acquired deformities, the logistic regression models did not converge, precluding calculation of adjusted ORs. Unadjusted ORs for these conditions are presented in Table 4.

## 4 Discussion

This study comprehensively analyzed the comorbidities of 1,003 patients diagnosed with HS, focusing on the most common chronic diseases and those most strongly associated with HS, irrespective of their overall frequency.

Our finding of a higher HS prevalence in women (52% increased likelihood) aligns with previous epidemiological data reporting a predominance in women, with female:male ration of approximately 3:1 (3, 27). While the reasons for this sex bias are not fully understood, exploring intrinsic and extrinsic sex-biased immunological factors, such as the microbiome and genetics, in related autoimmune conditions may offer insights into the underlying mechanisms (3, 27).

The observed HS prevalence of 0.10% in our study is lower than some global estimates, which range up to 0.40% and are often higher in clinical (1.7%) than population-based samples (0.3%) (4). This variation likely reflects differences in diagnostic criteria, study methodologies, and the populations studied, highlighting the challenges in comparing prevalence estimates across different studies (4). No significant association between HS prevalence and the percentage of women in each study has been reported to date (4).

Our observation of a lower HS prevalence among Spanish individuals compared to Sub-Saharan Africans, Latin Americans, and North Africans is consistent with previous research documenting racial and ethnic disparities in HS: previous studies have reported highest rates in African Americans (1.30%) and lower prevalence in Hispanics (0.07%) (28). The comparable rates found in Asians and Spaniards is also consistent with the relatively low prevalence reported in among Asians (28). However, variations in racial and ethnic classifications across studies complicate direct comparisons.

Consistent with previous research, our study found a higher prevalence of HS in the most deprived areas (Q4) (aOR 1.43, 95% CI 1.21–1.69), suggesting an association between lower socioeconomic status and HS (29, 30). This may be related to lifestyle factors such as diet, smoking, and access to healthcare resources (30). Current smoking status has a higher odds ratio for HS compared with controls without HS, with an OR of 4.26 (31). Approximately one-third of HS patients in our study had a documented history of smoking, which, while potentially an underestimate due to underreporting, falls within the range reported in other studies (40–92%) (31, 32).

To comprehensively understand the comorbidity profile in HS patients, we employed a two-step strategy. First, we outlined the most common comorbidities regardless of their relationship with HS. Next, we highlighted comorbidities with an increased probability of occurring in individuals diagnosed with HS. The interplay between HS and these comorbidities is complex and may involve causal, resulting, or shared etiological factors. Our study supports previous research, revealing that HS patients frequently experience multimorbidity, with almost three in four presenting with multiple diseases and symptoms of different origins.

Previous studies have shown a correlation between HS and obesity. Rates among patients with HS range from 12 to 88%, depending on the specific population studied (31, 33). A meta-analysis across Asia, Europe, and the United States involving 5,878 HS patients and over 13 million controls found that individuals with HS are four times more likely to be obese compared with the general population (pooled OR, 4.02 [2.66–6.06]) (34). In addition, obesity can exacerbate dyslipidemia. Several studies have reported a significant association between HS and lipid abnormalities, including hyperlipidemia and decreased high-density lipoprotein (HDL) cholesterol levels. The largest cross-sectional study of 3,207 HS patients and 6,412 controls found a significantly higher risk of hyperlipidemia in the HS group versus the control population (OR 1.17) (35). A case–control study also reported a significant association between HS and decreased HDL cholesterol levels in both hospital-and population-based HS patients (14).

Type 2 diabetes is a comorbidity of HS: a meta-analysis of 12 studies revealed a notably higher proportion of DM in HS cases than in non-HS healthy controls (16.1% vs. 15.7%), and a 2.17-fold higher risk of HS in patients with DM (8). The prevalence of DM in HS patients in out cohort was lower than that reported in other studies.

HS frequently impacts other vital organ systems, notably the digestive and musculoskeletal systems. While our study did not find significant associations with IBD, it is crucial to acknowledge the complex relationship between HS and conditions like Crohn’s disease and ulcerative colitis. Both HS and IBD are chronic inflammatory conditions predominantly affecting young individuals, and they are thought to share common genetic and environmental factors (16). The challenge in distinguishing between perineal manifestations of both diseases can complicate epidemiological findings. Nevertheless, numerous studies have highlighted a concurrent occurrence of HS and IBD, particularly with Crohn’s disease (36). Reported prevalence figures vary widely, ranging from 1.2 to 23%, depending on the specific populations and methodologies employed (16). For instance, two case–control studies revealed a 2.16- to 10-fold increased likelihood of IBD (encompassing both Crohn’s disease and ulcerative colitis) in individuals with HS (37). Furthermore, a separate cohort study reported a 5.6-fold elevated risk of developing IBD in patients with HS (37).

Musculoskeletal comorbidities represent a significant burden for HS patients. Inflammatory forms of arthritis, including rheumatoid arthritis, spondyloarthritis, and psoriatic arthritis, are well-documented associations. Previous research in HS patients indicates prevalences of 3.6% (OR 2.5; 95% IC 1.6–3.9) for rheumatoid arthritis, 9.1% (3.3; 2.0–5.4) for spondyloarthritis, and 4.9% (3.1; 1.8–5.3) for psoriatic arthritis (17, 18). In our cohort, we observed an association between HS and spondylosis, which may represent a broader category encompassing various forms of inflammatory arthritis, albeit with a lower observed odds ratio than reported in some prior studies (17, 18).

The association between HS and an increased risk of cardiovascular disease is a critical area of concern. Establishing a direct causal link between HS and cardiovascular conditions is challenging, primarily because HS frequently co-occurs with intermediate risk factors such as obesity and dyslipidemia, which themselves can independently alter cardiovascular risk (10, 12, 14). Hypertension, a well-established risk factor for cardiovascular diseases, is positively associated with HS. Our study population revealed a significant association between hypertension and HS, with a prevalence of 19.74% (aOR 1.26; CI 95% 1.05–1.52). However, we observed no significant association with cerebrovascular disease or acute myocardial infarction. Studies conducted in populations similar to ours have reported links between HS and comorbidities prevalent within those groups (11, 12, 38, 39). These divergent findings prompt further inquiry into whether our population exhibits distinct characteristics, or if variations in statistical adjustments might account for these differences.

Neuropsychiatric comorbidities are highly prevalent in HS patients and significantly contribute to their overall disease burden. Conditions such as depression, anxiety, and alcoholism have consistently shown a positive relationship with HS in existing literature, both in terms of occurrence and odds ratios (19–22). However, the reported prevalence rates for these conditions tend to fluctuate notably based on the study type and the specific screening methods employed. In our cohort, rates of related comorbidities including alcohol-related disorders, sexual disorders, anxiety disorders, and depression/mood disorders were slightly lower than those reported previously (21). This variability may be partially explained by the fact that our data were derived from diagnoses made by patients’ physicians, rather than from proactive screening initiatives.

Intriguingly, the neuropsychiatric comorbidity most strongly associated with HS in our study was schizophrenia and other psychotic disorders (aOR 2.17; 95% CI 1.16–4.06). This aligns with the findings of a systematic review and meta-analysis in which the likelihood of having schizophrenia was significantly higher in HS patients than control groups (OR 1.66, 95% CI: 1.53–1.79) (22). Chronic inflammation is posited as a key mechanism underlying the relationship between HS and schizophrenia, given that elevated levels of proinflammatory cytokines have been detected in HS skin lesions, as well as in the serum and cerebrospinal fluid of schizophrenia patients (22). Further supporting this connection, a large study in Finland identified a significant link between HS and schizophrenia, reporting a prevalence of 2.4% in HS patients, which is notably higher than rates observed both in psoriasis patients and the general control population (40).

Several studies have demonstrated a significant association between HS and acne vulgaris, particularly in younger patients (41, 42). Both conditions share common pathophysiological mechanisms, including follicular occlusion and neutrophilic inflammation, suggesting a shared pathogenic basis (42). The prevalence of acne among patients with HS is estimated at 15–20%, with higher rates observed in early-onset HS or syndromic phenotypes (41). In our study, acne was included under the broader coding category of “skin and subcutaneous tissue infections.” This categorization prevented us from establishing the specific prevalence of acne in HS patients. However, the overall prevalence of cutaneous infections in this group was 6.18%, indicating a significant association (aOR 3.32; 95% CI 2.56–4.30), and acne constitutes an important component of this category. This relationship is clinically important, both diagnostically and therapeutically. Azithromycin, a macrolide with anti-inflammatory properties, has shown efficacy in the treatment of acne in adolescents and may offer therapeutic benefits in HS owing to its immunomodulatory and antimicrobial effects (43). Similarly, tetracyclines such as doxycycline and minocycline, widely used in acne management, have shown clinical effectiveness in mild-to-moderate HS through their dual anti-inflammatory and antimicrobial actions (42). These therapeutic parallels underscore the importance of considering acne not only as a frequent comorbidity of HS but also as a potential avenue for shared treatment strategies.

A relationship between HS and neoplasms has been previously described. Recent studies report that patients with HS have an increased overall cancer risk and specifically an elevated risk of squamous cell carcinoma (44). In line with that study, we observed a prevalence of neoplasms of 5.88% in HS patients, but detected no significant association.

Our findings have important implications for the understanding of HS-related comorbidities, and highlight opportunities to refine HS management and improve patient well-being. Nonetheless, it should be noted that correlation does not imply causation, and thus caution is advised when interpreting these findings. While certain associations reported here align with those of earlier studies, others are novel and may necessitate further exploration to elucidate the underlying biological mechanisms.

The main strength of our research stems from the extensive, population-based approach applied, encompassing nearly all HS diagnoses within the reference area. Moreover, our comprehensive analysis of HS-related comorbidities involved a thorough examination of virtually all chronic conditions diagnosed in both primary and hospital care, and was not limited to the most prevalent or pertinent comorbidities. The utilization of Electronic Health Records (EHRs) ensured reliability of the data, which underwent continual quality control checks. However, it should be noted that clinical data were not clinically validated and were dependent on registered diagnostic codes, which may not fully represent the actual clinical status of patients. Another limitation of our study was its retrospective cross-sectional design, which inherently prevents determination of the sequential appearance of diseases, thereby impeding the establishment of cause-and-effect relationships between HS and its comorbidities. Moreover, some relevant variables (e.g., HS severity or staging, genetic information, social variables, patient-reported outcomes) were unavailable. Other variables, such as smoking status and obesity, were clearly underreported, precluding their inclusion in adjusted models. Finally, the grouping of the original diagnostic codes into Clinical Classifications Software (CCS) categories, while improving reproducibility and comparability, could potentially hamper the clinical interpretation of results due to the non-specificity of some diagnostic categories.

## 5 Conclusion

This large-scale, population-based study provides a comprehensive overview of the comorbidity burden associated with HS. Our findings confirm that HS is not merely a dermatologic condition, but also a systemic disease frequently associated with multiple chronic disorders across metabolic, cardiovascular, neuropsychiatric, musculoskeletal, and infectious domains. Nearly three out of four patients with HS experienced multimorbidity, highlighting the importance of early and integrated management. The strong associations identified between HS and conditions such as obesity, schizophrenia, skin infections, and white blood cell disorders emphasize the need for multidisciplinary care strategies and routine screening for comorbidities. These insights can inform clinical practice and policy by guiding risk stratification, surveillance, and resource allocation. Future longitudinal research is warranted to clarify temporal relationships and underlying mechanisms linking HS to its associated conditions.

## Data Availability

The raw data supporting the conclusions of this article will be made available by the authors, without undue reservation.
